# CCR5 Haplotypes and Mother-to-Child HIV Transmission in Malawi

**DOI:** 10.1371/journal.pone.0000838

**Published:** 2007-09-05

**Authors:** Bonnie R. Pedersen, Deborah Kamwendo, Melinda Blood, Victor Mwapasa, Malcolm Molyneux, Kari North, Stephen J. Rogerson, Peter Zimmerman, Steven R. Meshnick

**Affiliations:** 1 University of North Carolina, Chapel Hill, North Carolina, United States of America; 2 Case Western Reserve University Medical School, Cleveland, Ohio, United States of America; 3 University of Malawi College of Medicine, Blantyre, Malawi; 4 University of Melbourne, Melbourne, Australia; University of New South Wales, Australia

## Abstract

**Background:**

CCR5 and CCR2 gene polymorphisms (SNPs) have been associated with protection against HIV transmission in adults and with delayed progression to AIDS. The CCR5 Δ32 deletion and SNP -2459G are associated with reduced expression of the CCR5 protein.

**Methodology/Principal Findings:**

We investigated the association between infant CCR2/CCR5 diplotype and HIV mother to child transmission (MTCT) in Malawi. Blood samples from infants (n = 552) of HIV positive women who received nevirapine were genotyped using a post-PCR multiplex ligase detection reaction and haplotypes were identified based on 8 CCR2/CCR5 SNPs and the open reading frame 32 base pair deletion. Following verification of Hardy-Weinberg equilibrium, log linear regression was performed to examine the association between mutations and MTCT. Overall, protection against MTCT was weakly associated with two CCR5 SNPs, -2459G (Risk ratio [RR], 0.78; confidence interval [CI], 0.54–1.12), and the linked CCR5 -2135T (RR, 0.78; CI, 0.54–1.13). No child carried the CCR5 Δ32 SNP. Maternal Viral Load (MVL) was found to be an effect measure modifier. Among mothers with low MVL, statistically significant protection against MTCT was observed for -2459G (RR, 0.50; CI, 0.27–0.91), and -2135T (RR, 0.51; CI, 0.28–0.92). Statistically significant protection was not found at high MVL.

**Conclusions/Significance:**

Results from this study suggest that CCR5 SNPs -2459G and -2135T associated with reduced receptor expression protect against MTCT of HIV at low MVLs, whereas high MVLs may over-ride differences in coreceptor availability.

## Introduction

An estimated 600,000 children per year are infected with HIV-1, the majority of them by mother-to-child transmission (MTCT) [1]. Most of these children live in sub-Saharan Africa, where access to methods of preventing MTCT is still rare. MTCT can occur *in utero*, during labor and delivery, or through breastfeeding [2], [3].

There is evidence in children and adults that naturally occurring variations in HIV-1/chemokine receptors influence the risk of HIV-1 transmission and rate of HIV/AIDS disease progression. Genetic variations in chemokine receptor 5 (CCR5) and chemokine receptor 2 (CCR2) have been the focus of many recent HIV-1 transmission and disease progression studies [4].

Chemokine receptor 5 (CCR5) is an HIV co-receptor utilized by macrophage-tropic, or R5 strains of HIV-1 [5], [6], [7], [8], [9]. Homozygosity for a 32 base pair deletion (Δ32) in the open reading frame (ORF) has been shown to confer almost complete resistance to infection by R5 strains in adults [10], [11], [12], [13], [14]. This mutation has also been shown to be associated with reduced expression of CCR5 [15]. Similarly, several CCR5 promoter SNPs, principally -2459A→G and -2135C→T have been associated with decreased transmission or disease progression outcomes [16], [17], [18], [19], [20] as well as decreased infection and susceptibility to infection in vitro [21], [22]. A valine to isoleucine substitution at codon 64 in the CCR2b gene, in linkage disequilibrium with regions of the CCR5 promoter [23], is associated with delayed disease progression in some, though not all studies [23], [24], [25], [26], [27], [28].

**Figure 1:**
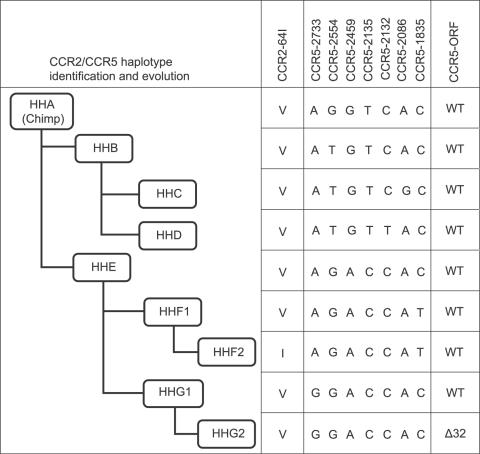
CCR2/CCR5 Haplotypes and abridged phylogenetic tree. Haplotypes were constructed based on the evolution of linked CCR2 and CCR5 mutations, including a valine to isoleucine substitution at codon 64 in the CCR2b gene (CCR2-64I), CCR5 mutations -2733A→G, -2554 G→T, -2459A→G, -2135C→T, -2132C→T, -2086A→G, -1835C→T, and the delta-32 deletion in the open reading frame of CCR5 (CCR5-ORF).

Because the various mutations in CCR2 and CCR5 are in linkage disequilibrium, human haplotypes A-E, F*1, F*2, G*1, and G*2 have been defined [29] (Figure 1).

Only a handful of studies have looked at CCR5 haplotype and susceptibility to MTCT. Haplotype D homozygosity was associated with increased susceptibility to MTCT [18] as was both haplotype E homozyogosity and heterozygosity [30]. A more recent study found a protective effect against MTCT of homozygosity for the G2 haplotype (Δ32) but only among children of mothers with lower viral loads [31]. Thus, CCR5 haplotypes appear to affect susceptibility to MTCT in a complex manner.

Here we studied a cohort of HIV positive women in Malawi to determine whether individual CCR2/CCR5 haplotypes are associated with perinatal transmission of HIV-1. Although mothers received nevirapine prior to delivery, approximately 20% of infants became HIV infected [32]. We further investigated whether there was an interaction between maternal viral load and polymorphisms in determining a child's relative risk of HIV acquisition.

## Methods

### Data Collection

We conducted ancillary data analysis of a larger completed cohort study described by Mwapasa et al, 2004 [33]. The original cohort study was recruited from December 2000 to June 2002 at Queen Elizabeth Central Hospital, Blanytre, Malawi. The estimated population in Blantyre at the time of the study was 950,000, 40% of whom lived in urban or periurban areas [33]. Study eligibility was determined among pregnant women attending the antenatal ward, after completing medical and obstetric assessment. Women in active labor, living outside of Blantyre, less than 15 years of age, with hypertension, or with altered consciousness were excluded. Consenting HIV positive women received nevirapine prior to delivery as well as post-HIV test counseling. Malaria infected women were treated with sulfadoxine pyrimethamine or oral quinine [33]. Infants of HIV-infected mothers received a single dose of nevirapine at birth. Infant whole blood for HIV testing and genotyping was collected at birth and at follow-up visits scheduled 6 and 12 weeks after delivery.

### Laboratory Methods

Maternal HIV status was established by two concordant HIV rapid tests (Determine®, Abbott Laboratories, Abbott Park, IL, USA, and Serocard®, Trinity BioTech, Dublin, Ireland). Infant and maternal DNA was extracted from whole blood using the QiAMP DNA Extraction Kit (Qiagen, Helden, Germany). Infant HIV status at birth, 6 and 12 weeks was determined by real time PCR [33]. Maternal HIV-1 RNA concentration measurements were performed using the Roche Amplicor HIV-1 Monitor® Test, version 1.5 (Roche Diagnostics, Branchburg, NJ). CD4 count was determined by FACSCount (BD, San Jose, CA).

Detection of the CCR5 ORF Δ32 by analysis of amplified fragment length polymorphisms was performed as previously described [34]. Infant genotype at CCR2-64V/I and CCR5 promoter SNPs –2733A/G, -2554G/T, -2459A/G, -2135C/T, -2132C/T, -2086A/G, -1835C/T was determined using a multiplex ligase detection reaction (LDR) based method with flow cytometric technology [35]. Briefly, a 1,118 base pair fragment of the CCR5 promoter containing the seven promoter SNPs and a 327 base pair fragment in the CCR2 ORF were PCR-amplified. The amplicon was then probed with two primers, an upstream allele specific primer with a unique 24 nucleotide FlexMAP™TAG sequence extension (Luminex® Corporation, Austin, TX) and a downstream 5′ phosphorylated/3′ biotinylated conserved sequence primer. Following allele specific hybridization, the primers were ligated. Ligation products were hybridized with fluorescent bead-labelled anti-TAG probes, and the 3′ biotin group labeled with phycoerythrin (PE). Mean fluorescence intensity of the allele-specific LDR:bead-labelled anti-TAG hybrid complexes was read on a BioPlex array reader (Bio-Rad Laboratories, Hercules, CA) into allele specific channels and used to determine genotypes (Bruse et al in preparation).

The original cohort study was approved by the Malawi College of Medicine Research and Ethics Committee and by the review boards at the University of Michigan and the University of North Carolina at Chapel Hill [33].

### Statistical Analysis

CCR2/CCR5 SNPs were tested for Hardy-Weinberg Equilibrium and Fisher's Exact Test was used to estimate pair-wise associations between MTCT and mutations or haplotypes, as well as the association between MTCT and maternal CD4 count, maternal viral load, maternal age, peripheral malaria parasite density, and mode of delivery (vaginal or caesarian).

Log-linear regression was performed to determine the association between each SNP and haplotype with MTCT. Due to the rarity of some SNPs, 3 category genotypes (heterozygotes and homozygous variant genotypes compared separately to the homozygous wild type referent genotype) as well as the presence/absence of the variant allele (hetero- and homozygous variants combined as one group and compared to the referent) were examined. Regression models were adjusted for all covariates used in the imputation of log MVL. Adjustment for multiple comparisons was made using the Bonferroni correction. Because log MVL is a strong determinant of MTCT, effect measure modification by MVL was examined by comparing results at below and above the median value of log MVL (4.57).

The Likelihood Ratio Test (LRT) was used to test for homogeneity of the associations between MTCT and SNP/haplotype, across MVL categories.

These statistical analyses were performed in SAS version 9.1.3 (SAS, Cary, NC).

Three MTCT outcomes were examined: infants infected via intrauterine transmission (IU), identified by a positive HIV PCR result at birth; infants infected via intrapartum transmission (IP), identified by first positive PCR result at six weeks after birth; and infants infected via peripartum transmission (PP), identified at week 12 of follow-up. A cumulative HIV transmission variable was created to represent HIV infection at any follow-up time in the study. To assess the consistency of our findings, sensitivity analyses were performed by modeling the outcomes IU, IP, PP, and cumulative transmission, in four separate models.

As the study progressed, it became too expensive to continue to determine maternal HIV viral load for each mother ($40/person), resulting in incomplete data for the last 22% of women to enroll in the study. To address this issue, we investigated multiple imputation for the missing maternal viral loads, using Markov Chain Monte Carlo simulation [36], [37], [38]. To meet assumptions of normality for imputation models, MVL was log-transformed. Maternal CD4 count was found to be the only variable predictive of the missingness of MVL and of MVL values; thus, it was the only variable used in the imputation models. Of the genotyped individuals, subjects missing data on both log MVL and maternal CD4 count were excluded (2%), giving a total analysis sample size of 529. Imputation models were performed in STATA version 9 use ICE and MICOMBINE commands.

## Results

### Demographics

552 infants of HIV positive women provided a blood sample at birth. 381 infants were captured at 6 weeks (69%) and 335 infants were captured at 12 weeks (61%). A total of 101 became HIV positive over the follow-up period (Table 1). Log transformed HIV viral load was available for 402 mothers (mean = 4.57, SD = 0.72) and after imputation, an additional 127 values were obtained (overall mean = 4.67, SD = 0.66).

**Table 1:** Frequency and HIV status of infants captured through follow-up

Status N (%)	At Birth	6 weeks*	12 weeks	Cumulative†
HIV negative	501 (90.8)	340 (89.2)	285 (85.1)	451 (81.7)
HIV positive	51 (9.2)	41 (10.8)	50 (14.9)	101 (18.3)
Total captured	552	381	335	552
Missing	0	172	218	0

* HIV status at 6 weeks and at 12 weeks includes incident and prevalent cases of HIV. Infants who missed week 6 and returned at week 12 or infants present at week 6 but lost at week 12 were included in the summary.

† Cumulative follow-up includes all infants who were ever HIV positive, regardless of loss to follow-up at one or more of the follow-up periods.

### Allelic Frequencies of Haplotypes and SNPs

**Figure 2:**
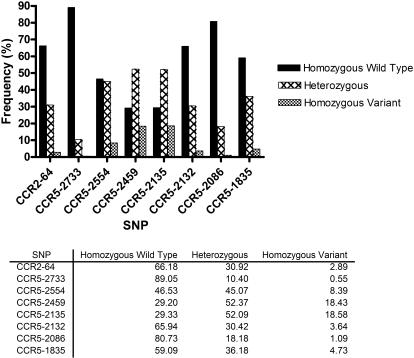
Genotype Frequencies. Infant genotype frequencies were calculated for all eligible births. Wild type and variant alleles correspond to the evolutionary history outlined in Figure 1.

**Figure 3:**
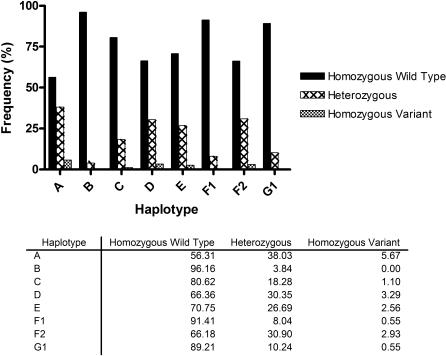
Haplotype Frequencies. Infant haplotype frequencies were calculated for all eligible births.

Among all eligible births for the study, the most common haplotypes were HHA, HHD, HHE, and HHF_2_, with allelic frequencies of 0.44, 0.34, 0.29 and 0.34, respectively. A lower frequency of haplotypes HHB (0.04) and HHF1 (0.09) was observed and HHG_2_ (bearing the Δ32 mutation) was absent (Figure 2). Allele frequencies for CCR2/CCR5 SNPs are summarized in Figure 3.

Because of the rarity of some SNPs, the homozygous mutant genotypes and heterozygotes were combined into one category referred to as “carriers” of the mutation of interest, which was compared to non-carriers of the mutation in regression models (Table 2).

**Table 2:** Association between Haplotype/SNP and MTCT

Haplotype/SNP	Carrier Status*	All Eligible Births N (%)	MTCT Births N (%)	RR (95% CI)†
A	− −	308 (56.3)	54 (53.5)	1.12 (0.78, 1.59)
	++/+−	239 (43.7)	47 (46.5)	
B	− −	526 (96.2)	94 (93.1)	1.96 (1.05, 3.66)
	++/+−	21 (3.8)	7 (6.9)	
C	− −	441 (80.6)	86 (85.2)	0.72 (0.43, 1.20)
	++/+−	106 (19.4)	15 (14.8)	
D	− −	363 (66.4)	62 (61.4)	1.24 (0.86, 1.77)
	++/+−	184 (33.6)	39 (38.6)	
E	− −	387 (70.8)	72 (71.3)	0.97 (0.66, 1.43)
	++/+−	160 (29.2)	29 (28.7)	
F1	− −	500 (91.4)	96 (95.1)	0.55 (0.24, 1.30)
	++/+−	47 (8.6)	5 (4.9)	
F2	− −	362 (66.2)	68 (67.3)	0.95 (0.66, 1.39)
	++/+−	185 (33.8)	33 (32.7)	
G1	− −	488 (89.2)	93 (92.1)	0.71 (0.36, 1.39)
	++/+−	59 (10.8)	8 (7.9)	
CCR2-64V→I	− −	366 (66.2)	67 (66.3)	1.00 (0.69, 1.45)
	++/+−	187 (33.8)	34 (33.7)	
CCR5-2733A→G	− −	488 (89.0)	93 (92.1)	0.70 (0.36, 1.36)
	++/+−	60 (11.0)	8 (7.9)	
CCR5-2554G→T	− −	255 (46.5)	43 (42.6)	1.18 (0.82, 1.68)
	++/+−	293 (53.5)	58 (57.4)	
CCR5-2459A→G	− −	160 (29.2)	35 (34.7)	0.78 (0.54, 1.12)
	++/+−	388 (70.8)	66 (65.3)	
CCR5-2135C→T	− −	161 (29.3)	35 (34.7)	0.78 (0.54, 1.13)
	++/+−	388 (70.7)	66 (65.3)	
CCR5-2132C→T	− −	362 (65.9)	61 (60.4)	1.27 (0.89, 1.81)
	++/+−	187 (34.1)	40 (39.6)	
CCR5-2086A→G	− −	444 (80.7)	86 (85.1)	0.73 (0.44, 1.21)
	++/+−	106 (19.3)	15 (14.9)	
CCR5-1835C→T	− −	325 (59.1)	64 (63.4)	0.84 (0.58, 1.21)
	++/+−	225 (40.9)	37 (36.6)	

* Non carriers of the haplotype or SNP were denoted as − − and carriers as ++/+−.

† The risk ratio (RR) was calculated from log linear regression, where non carriers (− −) were the referent. Maternal CD4 count, maternal age, and mode of delivery were not significantly associated with the SNPs/haplotypes or MTCT. Maternal viral load was significantly associated with MTCT but was not associated with SNPs or haplotypes. Therefore, these covariates were not included as confounders in the regression models.

### CCR5 polymorphism and MTCT

In bivariate analyses, only HHB appeared to increase the risk of MTCT (RR = 1.96, 95% CI = 1.05, 3.66). This relationship was not statistically significant after adjusting for multiple comparisons (*p* = 0.24). No other statistically significant associations between individual haplotypes and MTCT were observed, although the direction of haplotype associations was consistent with previous reports [18], [30] (Table 2). No association between CCR2-64I and MTCT was observed, with an RR of approximately 1.0. A protective effect was observed for CCR5 -2733G, -2459G, -2135T, -2086G, and -1835T, and an increase in the risk of MTCT was observed for CCR5 -2554T and -2132T, which are consistent in direction with previous reports, but the associations were not statistically significant (Table 2).

### Effect of CCR5 polymorphisms on MTCT at different MVL strata

**Figure 4:**
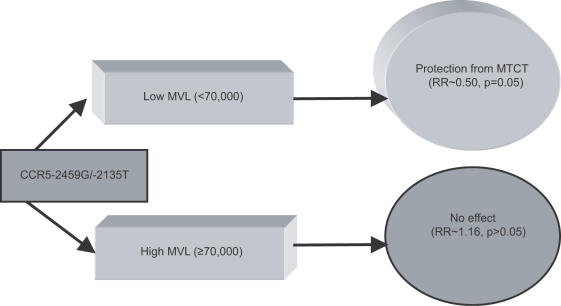
Effect Measure Modification by MVL. Carriers of CCR5 -2459G and -2135T with low MVL were protected from MTCT, whereas carriers of -2459G and -2135T with high MVL demonstrated no change in susceptibility to MTCT.

In order to investigate whether MVL may be an effect measure modifier similar to that described in Ometto et al. [31], we examined whether the associations between SNPs and MTCT were homogeneous, after stratifying at the median value of log MVL (4.57, which corresponds to a viral load of 37,000 copies/mL). Stratification revealed that associations between CCR5 -2459G, CCR5 -2135T and MTCT were not homogeneous across MVL strata (Table 3). This was confirmed by the likelihood ratio Chi-Square (1 df) which was 3.83 for CCR5-2459G (*p* = 0.05) and 3.60 for CCR5-2135T (*p* = 0.05). Thus, both SNPs appear to be protective against MTCT at low MVL, but not high MVL (Table 4, Figure 4).

**Table 3:** Likelihood Ratio Test (LRT) of Homogeneity* of the association across MVL categories

SNP	LRT	*p* value
CCR2-64V→I	0.38	0.54
CCR5-2733A→G	0.71	0.40
CCR5-2554G→T	0.049	0.83
CCR5-2459A→G	3.83	0.05
CCR5-2135C→T	3.60	0.05
CCR5-2132C→T	0.001	0.97
CCR5-2086A→G	2.02	0.15
CCR5-1835C→T	0.53	0.47

* The LRT is the likelihood ratio Chi-Square with 1 degree of freedom, calculated as the deviance of the full model minus the deviance of the reduced model, where the models differed by inclusion of the interaction term.

**Table 4:** Risk ratio modification of MTCT by MVL

	All Eligible Births N (%)	MTCT Births N (%)	RR (95% CI)*
Low MVL, CCR5 -2459G - -	77 (28.4)	16 (44.4)	1.0 (–)
Low MVL, CCR5 -2459G ++/+−	194 (71.6)	20 (55.6)	0.50 (0.27, 0.91)
High MVL, CCR5 -2459G - -	80 (29.4)	18 (28.1)	1.08 (0.60, 1.97)
High MVL, CCR5 -2459G ++/+−	192 (70.6)	46 (71.9)	1.16 (0.70, 1.92)
Low MVL, CCR5 -2135T - -	78 (28.8)	16 (44.4)	1.0 (–)
Low MVL, CCR5 -2135T ++/+−	193 (71.2)	20 (55.6)	0.51 (0.28, 0.92)
High MVL, CCR5 -2135T - -	80 (29.3)	18 (28.1)	1.10 (0.60, 1.99)
High MVL, CCR5 -2135T ++/+−	193 (70.7)	46 (71.9)	1.17 (0.71, 1.93)

* Non carriers of the SNP were denoted as − − and carriers as ++/+−.

† The risk ratio (RR) was calculated from log linear regression, where non carriers (− −) were the referent.

## Discussion

CCR5 plays a major role in host susceptibility to HIV-1 infection [10], [34], [39]. In Blantyre, Malawi, among mothers with low viral loads, this study found a reduction in the risk of MTCT in babies bearing specific CCR5 variants. Reductions in the risk of MTCT were found for two linked CCR5 mutations, CCR5 -2459G and -2135T at low maternal viral loads.

The frequencies of CCR5 variants observed in our study are similar to those reported in MTCT studies of African-American populations, in particular the absence of the Δ32 mutation, the very low frequency of HHB, and the relatively higher minor allele frequency of CCR5 -2459G, -2135T, and -2132T [18], [27].

Carriage of CCR5 -2459G mutation has been associated with reduced density of CCR5 on CD14+ monocytes and lower levels of R5 HIV propagation when compared to CCR5 -2459A [21], [40]. Thus, as with the Δ32 mutation, reduced expression is correlated with protection against MTCT. The functional significance of -2459G is a key characteristic of this SNP in comparison to the other SNPs included in the haplotypes. Linkage disequilibrium between -2459G and -2135T may explain the similar findings for the two SNPs.

Our findings are somewhat different from 3 previous studies on MTCT. While we did not find a significant association between haplotype HHD homozygosity (bearing -2132T) and MTCT [18], like Kostrikis et al. we found a non-significant association suggesting that HHD was associated with increased risk of MTCT [18]. We did not observe an effect of HHE carriage or homozygosity on MTCT as seen in Argentinean children [30]. In contrast to John et al. who did not observe any influence of CCR5 gene polymorphism on MTCT in a Kenyan cohort, we observed a specific effect of reduced MTCT in association with CCR5 promoter polymorphisms characterized by reduced gene expression [41].

There are four explanations for these discordant results. First, our study was the only one to account for effect measure modification by MVL. Second, our study was the only one to be conducted in mother-infant pairs treated with nevirapine. Third, all four studies were done in different populations where contributions from other genetic and environmental cofactors could occur. Finally, all of the studies comprise relatively small numbers and conduct multiple comparisons, and could be underpowered to find important associations.

All women and their babies received nevirapine which would have reduced HIV inoculum size. Thus, nevirapine would likely cause an underestimate of the effect seen in the absence of treatment. There are many other determinants of transmission including obstetric factors and mother-to-child microtransfusion [42] that were not analyzed in our study but which should also have affected our findings in a non-differential manner.

Nearly all (98% at 6 weeks, 96% at 12 weeks) women in our study breast-fed. However, only 80% exclusively breast-fed at 6 weeks and only 46% exclusively breast-fed at 12 weeks. Exclusive breastfeeding at either 6 or 12 weeks was not associated with the main exposures (CCR5-2459G/-2135T) or the main outcome (MTCT) of interest (Pearson Chi-square p-values>0.05); thus was not viewed to be a potential confounder in the analysis.

To address the loss to follow-up in our study, we conducted sensitivity analyses investigating the association between SNP and MTCT at different times of follow-up in the study. Our results showed that the effects of CCR5-2459G and -2135T are consistent across follow-up (Table 5).

**Table 5:** Sensitivity analyses for SNP association with MTCT at different time points of follow-up

SNP	Model	Birth RR (95% CI)†	6 weeks RR (95% CI)	12 weeks RR (95% CI)	Cumulative RR (95% CI)
CCR5-2459A→G	Overall	0.76 (0.44, 1.31)	0.93 (0.61, 1.44)	0.70 (0.47, 1.04)	1.00 (0.69, 1.45)
	Low MVL	0.14 (0.04, 0.51)	0.38 (0.14, 1.00)	0.29 (0.11, 0.80)	0.70 (0.36, 1.36)
	High MVL	1.38 (0.61, 3.10)	1.45 (0.78, 2.73)	1.03 (0.60, 1.80)	1.18 (0.82, 1.68)
CCR5-2135C→T	Overall	0.76 (0.45, 1.32)	0.94 (0.61, 1.45)	0.70 (0.47, 1.05)	0.78 (0.54, 1.12)
	Low MVL	0.14 (0.03, 0.52)	0.39 (0.15, 1.04)	0.30 (0.11, 0.83)	0.78 (0.54, 1.13)
	High MVL	1.38 (0.61, 3.10)	1.46 (0.78, 2.73)	1.03 (0.60, 1.80)	1.27 (0.89, 1.81)

† The risk ratio (RR) was calculated from log linear regression, where non carriers (− −) were the referent.

To address the fact that we were missing 22% of MVL data, we conducted multiple imputation of MVL. Because only CD4count was used in the imputation models, this was actually single imputation. This approach was viewed to be more accurate than including variables that have no biological or statistical significance for predicting MVL. Our imputation results show that CCR5-2459G and -2135T are consistently associated with MTCT both overall and after dichotomizing by MVL (Table 6). Although a complete dataset is preferable, the missing data in our study did not appear to be problematic for accurate interpretation of results.

**Table 6:** Imputation Results for 5 imputations (N = 529 per imputation)

SNP	Overall RR (95% CI)†	Low MVL RR (95% CI)	High MVL RR (95% CI)
CCR2-64V→I	1.03 (0.65, 1.62)	0.76 (0.26, 2.21)	1.23 (0.67, 2.27)
CCR5-2733A→G	0.64 (0.30, 1.40)	0.56 (0.09, 3.37)	0.69 (0.24, 1.92)
CCR5-2554G→T	1.25 (0.81, 1.94)	1.59 (0.63, 4.06)	1.16 (0.65, 2.04)
CCR5-2459A→G	0.74 (0.47, 1.17)	0.37 (0.16, 0.88)	1.11 (0.60, 2.04)
CCR5-2135C→T	0.75 (0.48, 1.19)	0.38 (0.16, 0.90)	1.13 (0.62, 2.07)
CCR5-2132C→T	1.49 (0.96, 2.30)	1.59(0.67, 3.75)	1.54 (0.86, 2.74)
CCR5-2086A→G	0.68 (0.37, 1.23)	1.02 (0.33, 3.14)	0.52 (0.23, 1.18)
CCR5-1835C→T	0.83 (0.53, 1.29)	0.58 (0.21, 1.56)	1.05 (0.58, 1.91)

† The risk ratio (RR) was calculated from log linear regression, where non carriers (− −) were the referent.

The generalizability and replicability of our study may be limited because the median value of log MVL (4.57) was used to create the dichotomous variable. We could have used a value of 4, which corresponds to a viral load of 10,000 copies/µL, a more commonly used value in the literature. However, our cut point was determined as a key component to a priori set hypotheses.

Because log-linear regression models using imputed data are considered to be experimental in STATA (version 9), we presented odds ratios from logistic regression models to estimate risk ratios. This may have biased our results away from the null, as odds ratios from logistic regression generally overestimate the relative risks from log-linear regression. However, we compared the results of the experimental log-linear and logistic imputation models and found similar results (data not shown).

In conclusion, this study characterized the role of infant CCR5 polymorphisms in susceptibility to MTCT in nevirapine-treated African mother-infant pairs. Our results suggest a protective effect of CCR5 -2459G and -2135T among infants of mothers with low maternal viral load, which might be due to a reduction in the expression of the CCR5 receptor. The influence of MVL on this effect suggests that there may be a threshold in the ratio of virus to receptor above which changes in the receptor concentration have no effect on viral infectivity. Although our study was not of heterosexual transmission, it may have implications for microbicides designed to block access to CCR5 [43]. If a threshold exists for receptor access by virus, there will also be a threshold for blocking receptor access by virus. Ensuring that a CCR5-blocking microbicide is present at a concentration that will not be overwhelmed by viral load is critical. These findings are also applicable to antiretroviral drugs such as CCR5 inhibitors [44] and chemoprophylaxis in high risk populations, where delivery mechanisms differ from those of vaginal microbicides. Continuing studies on the genetics of MTCT will provide better insight into the pathophysiology and prevention of HIV-1 MTCT with potential applications to other modes of transmission.
